# Impact of outcome adjudication in kidney disease trials: observations from the Study of Heart and Renal Protection (SHARP)

**DOI:** 10.1016/j.ekir.2023.05.008

**Published:** 2023-08

**Authors:** William G. Herrington, Charlie Harper, Natalie Staplin, Richard Haynes, Jonathan Emberson, Christina Reith, Lai Seong Hooi, Adeera Levin, Christoph Wanner, Colin Baigent, Martin Landray, on behalf of the SHARP Collaborative Group

**Affiliations:** 1 Medical Research Council Population Health Research Unit at the University of Oxford, Nuffield Department of Population Health (NDPH), University of Oxford, UK; 2 Clinical Trial Service Unit and Epidemiological Studies Unit (CTSU), NDPH, University of Oxford, UK; 3 Oxford Kidney Unit, Oxford University Hospitals NHS Foundation Trust, Oxford, UK; 4 Hospital Sultanah Aminah, Johor Bahru, Malaysia; 5 University of British Columbia, Vancouver, Canada; 6 University Clinic of Würzburg, Würzburg, Germany

**Keywords:** clinical trials, dialysis, transplantation, chronic kidney disease, adjudication

## Abstract

**Introduction:**

We aimed to assess opportunities for trial streamlining and the scientific impact of adjudication on kidney and cardiovascular outcomes in CKD.

**Methods:**

We analysed the effects of adjudication of ~2100 maintenance kidney replacement therapy (KRT) and ~1300 major atherosclerotic events (MAEs) recorded in SHARP. We first compared outcome classification before versus after adjudication, and then re-ran randomised comparisons using pre-adjudicated follow-up data.

**Results:**

For maintenance KRT, adjudication had little impact with only 1% of events being refuted (28/2115). Consequently, randomised comparisons using pre-adjudication reports found almost identical results (pre-adjudication: simvastatin/ezetimibe 1038 vs placebo 1077; risk ratio [RR] 0.95, 95%CI 0.88–1.04; post-adjudicated: 1057 vs 1084; RR=0.97, 95%CI 0.89–1.05). For MAEs, about one-quarter of patient reports were refuted (324/1275 [25%]), and reviewing 3538 other potential vascular events and death reports identified only 194 additional MAEs. Nevertheless, randomised analyses using SHARP’s pre-adjudicated data alone found similar results to analyses based on adjudicated outcomes (pre-adjudication: 573 vs 702; RR=0.80, 95%CI 0.72-0.89; adjudicated: 526 vs 619; RR=0.83, 95%CI 0.74– 0.94), and also suggested refuted MAEs were likely to represent atherosclerotic disease (RR for refuted MAEs=0.80, 95%CI 0.65−1.00).

**Conclusions:**

These analyses provide three key insights. First, they provide a rationale for nephrology trials not to adjudicate maintenance KRT. Secondly, when an event that mimics an atherosclerotic outcome is not expected to be influenced by the treatment under study (e.g. heart failure), the aim of adjudicating atherosclerotic outcomes should be to remove such events. Lastly, restrictive definitions for the remaining suspected atherosclerotic outcomes may reduce statistical power.

## Introduction

Randomised trials are the cornerstone of evidence-based practice, but scientifically unjustified and costly procedures have led trials to become excessively complex. Clinical outcome adjudication is the process of verification of reported outcomes using source documents (e.g. medical notes) according to pre-specified diagnostic criteria by trained clinicians blind to study treatment allocation. It may offer reassurances about the reliability of a trial’s findings and have particular value for outcomes with complex presentations with multiple alternative diagnoses (e.g. heart failure), if a precise diagnosis is critical for trial interpretation (e.g. reliably distinguishing between ischaemic and haemorrhagic stroke in an anti-thrombotic trial where treatment effects are expected to differ qualitatively), or if reliability of the reporting source is unknown.^1^ However, clinical adjudication of cardiovascular (CV) outcomes in trials conducted in populations with low prevalence of chronic kidney disease (CKD) has been shown to have almost no meaningful effect on trials’ observed relative treatment effects.^2,3^ In addition the financial cost of adjudication, which may limit sample size or trial duration, adjudication could also conceivably harm a trial. For example, overly specific adjudication criteria may lead to “true” outcomes being refuted, reducing trial power (where not event driven). Adjudication could also conceivably distract the focus of recruiting sites and trial coordinating centres from procedures which are critical-to-quality, such as maximising adherence to allocated study treatment and completeness of follow-up^4,5^. As increasing complexity of conducting clinical trials threaten our ability to answer important future clinical questions,^6,7^ reviewing the scientific value of expensive time-consuming trial procedures, like adjudication, and fostering appropriate streamlining could help increase the number practice-changing trials in nephrology and improve current and future patients’ care.^5^

Recognising these issues, KDIGO recommended that kidney trials are tailored such that “adjudication methods to focus on those events in which adjudication may materially influence interpretation of the results”,^5^ and the International Society of Nephrology’s 1st International Consensus Meeting on Defining Kidney Failure in Clinical Trials concluded that “the role of event adjudication for kidney failure needs to be evaluated within the specific context of the study planned”.^8^ Patients with CKD are at increased risk of CV diseases as well as kidney failure,^9^ with documentation related to kidney and CV outcomes often collected and adjudicated in nephrology trials. We aimed to use data from the Study of Heart and Renal Protection (SHARP) to assess the reliability of kidney replacement therapy (KRT) and CV clinical outcomes reported by patients with CKD as compared to a gold standard of outcomes derived following adjudicated direct follow-up.

## Methods

SHARP’s design and results are reported elsewhere.^10,11^ In brief, SHARP assessed the effect of lowering LDL-cholesterol with simvastatin 20mg plus ezetimibe 10mg once daily versus matching placebo on the incidence of major atherosclerotic events (MAEs) among 9270 patients with CKD followed for a median follow-up of 4.9 years. MAEs were defined as non-fatal myocardial infarction or coronary death (referred to as major coronary events), non-haemorrhagic stroke (ischaemic and unknown subtypes combined), or arterial revascularisation (coronary or non-coronary, excluding dialysis access). End-stage kidney disease was defined as initiation of maintenance haemodialysis (defined as planned dialysis initiation or dialysis status for ≥3 months) or receipt of a kidney transplant, and is referred to as maintenance KRT throughout this manuscript.

The main method of follow-up was clinic-based systematic interviews of participants (or with relatives/clinicians in the event of death) every 6 months. Participants’ answers were recorded and coded in real-time directly into clinical terms on a computer-system by trained local research coordinators (usually nurses). Specific descriptive codes for initiation of maintenance haemodialysis and peritoneal dialysis were used, with temporary dialysis distinguished using the code “acute-on-chronic renal failure requiring dialysis”. Freetext descriptions, recoded by study clinicians at the central coordinating centre, were only used when local research coordinators were uncertain about an appropriate event code term. There was no requirement for direct review of reported events by a local physician investigators. All deaths, MAEs, potential MAEs (i.e. events terms which could represent a miscoded MAE, see Supplemental Table S1 for full details), and KRT events (maintenance or not) were then subject to clinical adjudication. The central coordinating centre requested documentation (e.g. hospital discharge summaries, death certificates, clinical investigation reports, vascular access procedures etc.) for each of these potential outcomes. All mentions of use of lipid-modifying drugs and laboratory measurements of lipids were redacted before documents were made available to nephrologist adjudicators blind to treatment allocation. They followed a standard operating procedure containing pre-specified diagnostic criteria. If, during the process of adjudicating one event, the adjudicator came across information about a potential clinical outcome that had not been reported, then they would create a new report in the study database. Adjudication documents were available in >90% of potential outcomes.

## Statistical methods

In this report, tabulations of pre-adjudication versus post-adjudication outcome categories (based on the first relevant event in each category) were constructed and the proportion of pre-adjudicated outcomes refuted by adjudication calculated. Agreement statistics were not presented as the two outcome datasets were not independent.^12^ Differences between event dates before and after adjudication were presented as categories, including: exact match, 1-7 days, 8-30, 31-90, 91-180, and >180 days. Randomised comparisons of the effect of allocation to study intervention using intention-to-treat time-to-first event log-rank methods were re-run, presenting rate ratios (RRs) with 95% confidence intervals (95%CI).^10,11,13,14^ These analyses were conducted for maintenance KRT and MAE outcomes based on pre-adjudication participant-reports and compared with the published post-adjudication outcome data.^10,11^ The 95%CI for the difference between RRs was estimated using bootstrap methods. For MAE, analyses were also performed using refuted, unrefuted, and “identified by adjudication” events only; and considering separately patients not on dialysis or on dialysis at randomisation. All analyses were conducted using SAS version 9.4 and R version 4.2.2.

## Results

### Maintenance KRT

Among the 6245 patients not on dialysis at randomisation, 2115 maintenance KRT events were reported, of which less than 1% (n=28) were refuted by adjudication (Table 1). Only one of the 504 reported kidney transplant events was refuted. Adjudicating 312 events reported as acute-on-chronic renal failure events requiring dialysis identified an additional 54 maintenance KRT events. Differences in reported dates of start of maintenance KRT between datasets of >30 days were uncommon (<7%, Supplementary Table S2).

**Figure 1:**
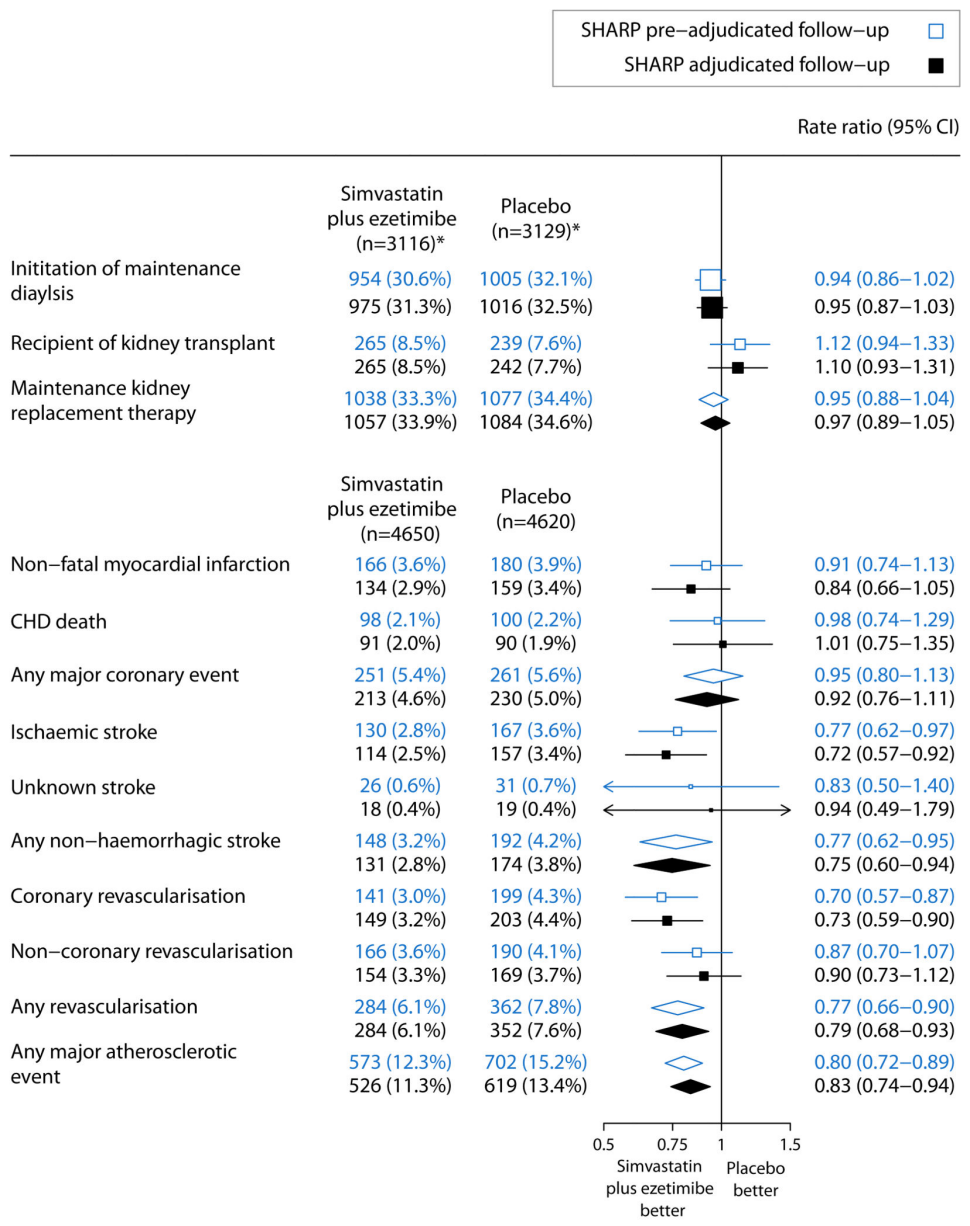
Effect of allocation to simvastatin/ezetimibe vs placebo on maintenance kidney replacement therapy and major atherosclerotic events using SHARP adjudicated and pre-adjudicated follow-up Log-rank methods were used to calculate the rate ratio and 95% confidence intervals. *Excluding those on maintenance dialysis at baseline. Risk ratio for the major atherosclerotic events refuted by adjudication was 0.80 (95% CI 0.65–1.00). CI=confidence interval. CHD=coronary heart disease.

Randomised analyses using pre-adjudication data showed that allocation to simvastatin/ezetimibe had no effect on risk of maintenance KRT (simvastatin/ezetimibe 1038 vs placebo 1077; risk ratio [RR] 0.95, 95%CI 0.88–1.04), almost identical to results from adjudicated direct follow-up (1057 vs 1084; RR=0.97, 95%CI 0.89–1.05; Figure 1). The 95%CI for the -0.01 difference between these RRs was -0.03 to 0.00. Similarly, RRs estimated from pre- and post-adjudication events were almost identical for the outcomes of initiation of maintenance dialysis and receipt of a kidney transplant considered separately.

### Major atherosclerotic events

Among the full 9270-participant SHARP cohort including patients on dialysis, 1275 first MAEs were reported, of which 25% (324/1275) were refuted by adjudication (Table 1). Among the components of the MAE outcome, major coronary events had the highest proportion of refuted events (214/512 [42%]), with coronary death reports (111/198 [56%]; Supplemental Table S3) being more likely to be refuted than non-fatal MI reports (132/346 [38%]). Non-haemorrhagic stroke reports (102/340 [30%]), and revascularisations (102/646 [16%]) were less likely to be refuted. Of the MAEs which were refuted, 70.6% led to hospitalisation versus 95.9% of unrefuted MAEs, but when a hospitalisation did result, median stay duration was similar (7 [interquartile range 2-14] versus 7 [4-15] days, respectively; Supplementary Table S4). After adjudicating 3538 other potentially relevant event reports that could represent a miscoded MAE (e.g. non-coronary CV deaths and all non-CV deaths, angina events, transient ischaemic attacks, coronary angiograms; see Supplementary Table S1 for the full list of terms), 194 additional first MAEs that had not been reported by sites were identified from collected adjudication source documents. These included 48 non-fatal myocardial infarctions and 58 CHD deaths (Table 1 footnote).

Reported MAEs were more likely to be refuted among patients on dialysis for non-fatal MIs (dialysis vs non-dialysis: 45% vs 34%) and coronary revascularisations (25% vs 10%) (Supplementary Table S5). Differences of >30 days for start dates of MAE between datasets were uncommon (11% of MAE; Supplementary Table S2).

**Figure 2:**
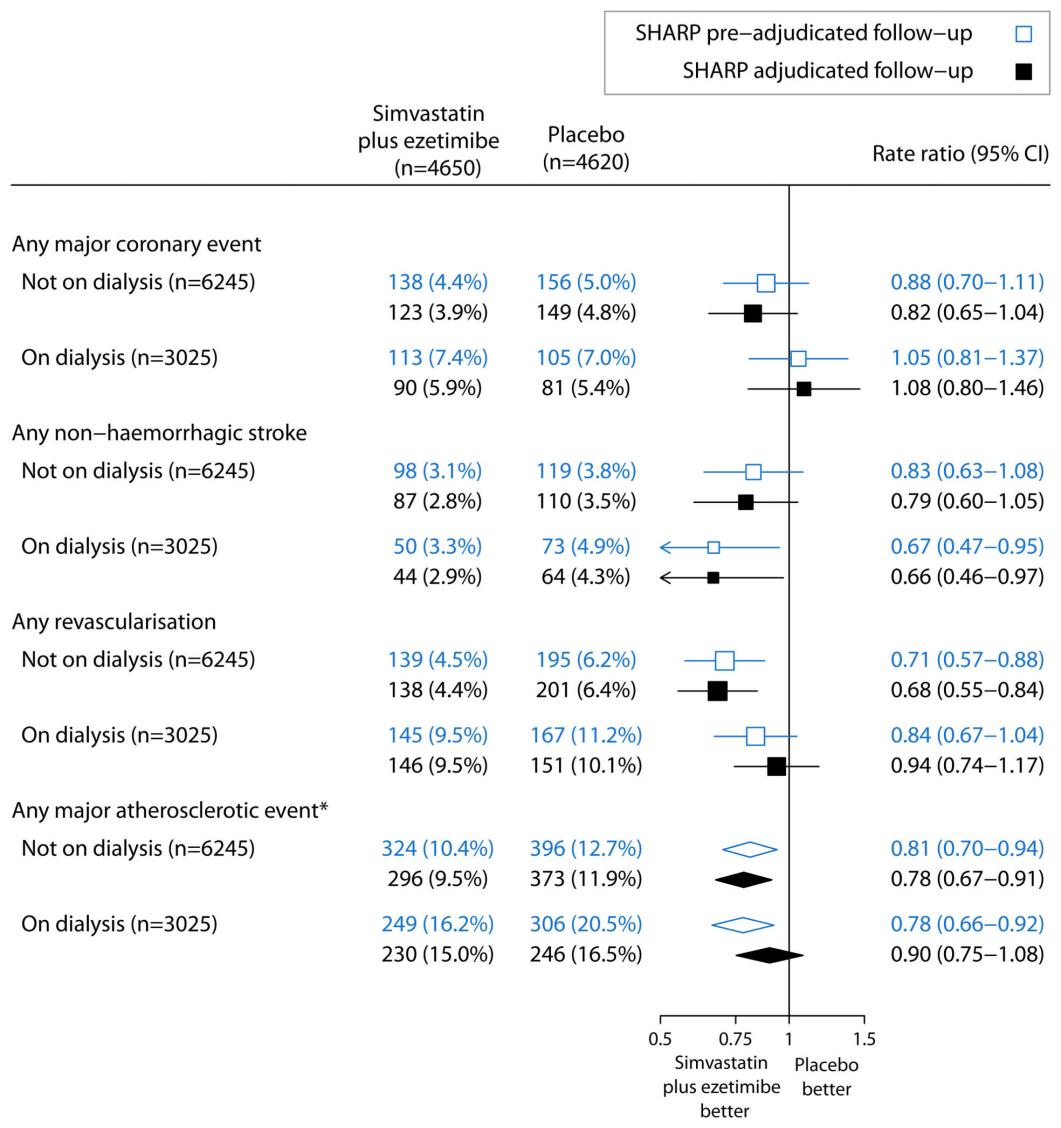
Effect of allocation to simvastatin/ezetimibe vs placebo on major atherosclerotic events using SHARP adjudicated and pre-adjudicated follow-up, by dialysis status Log-rank methods were used to calculate the rate ratio and 95% confidence intervals. *Heterogeneity between patients on dialysis and not on dialysis: $\chi \frac{2}{1}=1.35$ (p=0.24) for adjudicated follow-up and $\chi \frac{2}{1}=0.11$ (p=0.74) for pre-adjudicated follow-up. CI=confidence interval.

Analyses comparing the effects of allocation to simvastatin/ezetimibe versus placebo on pre-adjudication MAE reports demonstrated similar findings to those using adjudicated follow-up data (Figure 1). Allocation to simvastatin/ezetimibe reduced the risk of pre-adjudicated MAE by 20% (simvastatin/ezetimibe 573 vs placebo 702; RR=0.80, 95%CI 0.72-0.89), similar to the 17% reduction in risk of adjudicated MAE (526 vs 619; RR=0.83, 95%CI 0.74–0.94; difference between RRs -0.03, 95%CI -0.10, +0.03). RRs based on adjudicated and pre-adjudication follow-up were similar across the components of MAE. The RR for the effect of treatment allocation on the 324 MAEs refuted by adjudication was 0.80 (95%CI 0.65-1.00), and 1.02 (95%CI 0.77-1.35) for the 194 MAEs identified by adjudication of other potentially relevant event reports (Supplementary Table S4). In analyses comparing patients by baseline dialysis status, the heterogeneity test p values for the effect of simvastatin/ezetimibe versus placebo on MAE was 0.24 for adjudicated MAE, and 0.74 for pre-adjudicated MAE (Figure 2).

## Discussion

These analyses from the double-blind placebo-controlled SHARP trial found that information about events reported by participants to trained research coordinators - without review by local or central clinicians - provided consistent estimates of relative treatment effects for the outcomes of maintenance KRT and MAE. In particular, findings from pre-adjudication and post-adjudication outcome data were almost identical for kidney transplantation and very similar for initiation of maintenance dialysis and revascularisation procedures. They provide a particularly strong rationale for refining consensus conclusions regarding adjudication of kidney failure to support more streamlined approaches.^8^ These would ideally encourage not adjudicating KRT outcomes from adequately blinded trials in CKD populations, as the vast majority of events are correctly categorized when reported.

The presented SHARP observations based on populations from North America, Europe and Asia provide the largest and most generalisable experience of adjudicating KRT and CV outcomes in a CKD trial. The SHARP CV data also confirm that previous outcome adjudication experiences from diabetes and cardiology trials are relevant to CKD trials. SHARP results mirror those from the 15,480 participant ASCEND trial in diabetes^15^ and from 42 cardiology trials (including over 200,000 participants) which together have consistently reported that that relative treatment effects on CV outcomes were not materially changed by clinical adjudication.^2,3^ Future extension of the presented research using a similar approach to our recent analyses from the ASCEND trial^15^ is now indicated to assess if unadjudicated routinely collected hospital admission data from different regions of the world are sufficiently reliable to be used as the sole method of follow-up for KRT and CV outcomes in CKD trials.

SHARP data may provide a rationale for not requiring adjudication of MAEs. Despite the fact that substantial re-categorisation occurred during adjudication, this had no material impact on the RRs for confirmed, refuted and newly identified outcomes which were similar. In fact, it is possible that our approach to CV outcome adjudication may have reduced SHARP’s power to detect treatment effects, especially in subgroups. The similar sized RRs for the 1145 confirmed MAEs and the 324 refuted MAEs suggest that many of the CV events that did not fulfil the pre-specified criteria for confirming MAEs - the majority of which led to hospitalisation for several days - were prevented by lowering LDL-cholesterol and hence represented modifiable atherosclerotic disease. It was notable that agreement between pre- and post-adjudicated datasets was less strong for major coronary events than other MAE components (non-haemorrhagic stroke and revascularisation procedures). This may reflect the challenges of distinguishing between atherosclerotic and non-atherosclerotic cardiac events based on clinical findings in patients with advanced CKD.^16,17^ The corollary of these SHARP observations is that if CV outcome adjudication is deemed scientifically important in a CKD trial, then outcome definitions for major coronary events should allow for inclusion of atypical presentations and incomplete information. Avoiding overly strict and overly specific adjudication criteria could reduce the chances of “real” outcomes being refuted and maintain trial power. This may have been particularly true among patients on dialysis whose reported MAEs were more likely to be refuted. Both the relative and absolute effects of lowering LDL-cholesterol on MAEs in patients on dialysis may have been underestimated in SHARP (Figure 2).

In summary, these findings from SHARP lead to three key conclusions with practical implications for outcome adjudication in trials in CKD. First the data provide a rationale to stop adjudication of maintenance KRT in CKD progression trials as the vast majority were correctly classified on reporting. Secondly, when an event that mimics an atherosclerotic outcome is not expected to be influenced by the treatment under study (e.g. heart failure), the aim of adjudicating atherosclerotic outcomes should be to remove such events. Lastly restrictive definitions for the remaining suspected atherosclerotic outcomes may reduce statistical power.

## Supplementary Material

Supplementary materials

## Figures and Tables

**Table 1:** Tabulation of maintenance kidney replacement therapy and major atherosclerotic events before and after adjudication in SHARP

Outcome	Total events before adjudication	Unrefuted by adjudication	Refuted by adjudication	Identified by adjudication of other reported events	Total events after adjudication
Maintenance dialysis*	**1959**	1930 (99%)	29 (1%)	61	**1991**
Kidney transplant*	**504**	503 (>99%)	1 (<1%)	4	**507**
Maintenance kidney replacement therapy*	**2115**	**2087 (99%)**	**28(1%)**	**54**	**2141**
Non-fatal myocardial infarction	**346**	214 (62%)	132 (38%)	79	**293**
CHD death	**198**	87 (44%)	111 (56%)	94	**181**
**Any major coronary event**	**512**	**298 (58%)**	**214 (42%)**	**145**	**443**
Ischaemic stroke	**297**	210 (71%)	87 (29%)	61	**271**
Unknown stroke	57	6 (11%)	51 (89%)	31	**37**
**Any non-haemorrhagic stroke**	**340**	**238 (70%)**	**102 (30%)**	**67**	**305**
Coronary revascularisation	**340**	283 (83%)	57 (17%)	69	**352**
Non-coronary revascularisation	**356**	285 (80%)	71 (20%)	38	**323**
**Any revascularisation**	**646**	**544 (84%)**	**102 (16%)**	**92**	**636**
**Any major atherosclerotic event**	**1275**	**951 (75%)**	**324 (25%)**	**194**	**1145**

Percentages in parentheses are % of total number of SHARP participants with the outcome reported before adjudication. *Excluding those on maintenance dialysis at baseline. The 194 first major atherosclerotic events (MAEs) identified by adjudication of 3538 potential MAEs (i.e. events terms which could represent a miscoded MAE) included: 48 non-fatal myocardial infarctions, 58 CHD deaths, 36 ischaemic strokes, 19 unknown strokes, 12 coronary revascularisations, and 21 non-coronary revascularisations. See Supplementary Table S1 for a summary of the 3538 potential MAEs selected for adjudication. CHD = coronary heart disease.

## Data Availability

Access to the SHARP data reported in this manuscript is available according to the Nuffield Department of Population Health Data Access and Sharing Policy available at https://www.ndph.ox.ac.uk/about/data-access-policy.

## Lay summary

In 2003-2010, the SHARP trial tested the effects of cholesterol lowering in 9270 patients with chronic kidney disease. We have assessed the impact of clinician confirmation of the participant-reported outcomes of dialysis, transplantation, heart disease and stroke recorded during trial interviews. We found that clinician confirmation had little effect on the reliability of the trial’s results, and may have actually reduced its ability to detect true effects of cholesterol lowering on heart disease (particular among patients on dialysis). This provides a rationale to stop clinician confirmation of patient-reported kidney replacement therapy and simplify definitions used for defining heart disease.
