# Supplementary material for: Impact of Outcome Adjudication in Kidney Disease Trials: Observations From the Study of Heart and Renal Protection
Source: Kidney Int Rep. 2023 May 16;8(8):1489–95. doi: 10.1016/j.ekir.2023.05.008 (PMC7614871; doi:10.1016/j.ekir.2023.05.008)
Supplement: Supplementary File (PDF) [file mmc1.pdf]

Supplemental Materials

Impact of outcome adjudication in kidney disease trials: observations from the Study of Heart and Renal Protection (SHARP)

Supplementary Table S1: Cardiovascular events selected for adjudication to identify potential missed major atherosclerotic events in SHARP

Supplementary Table S2: Comparison of pre-adjudication and post-adjudication dates

Supplementary Table S3: Comparison of pre-adjudication and post-adjudication follow-up for deaths in SHARP

Supplementary Table S4: Fact of hospitalisation, duration of hospital stay, and effect of allocation to simvastatin/ezetimibe vs placebo on refuted, unrefuted and "identified by adjudication" major atherosclerotic events

Supplementary Table S5: Tabulation of major atherosclerotic events before and after adjudication in SHARP, by dialysis status

Stroke Statement Checklist

Supplementary Table S1: Cardiovascular events selected for adjudication to identify potential missed major atherosclerotic events in SHARP

Event category	Number of events before adjudication
**Non-coronary deaths**	
Non-coronary vascular deaths	677
Non-vascular deaths	1364
**Subtotal: Non-coronary deaths**	**2041**
**Cardiac related events**	
Hospitalisation with angina	306
Cardiac arrest	38
Ventricular tachycardia	19
Chest pain/tightness	182
Heart failure	324
Cor pulmonale/right heart failure	11
**Subtotal: Cardiac related events**	**880**
**Cerebrovascular related events**	
Haemorrhagic stroke	52
Subarachnoid haemorrhage	10
Spontaneous subdural haematoma	3
Transient ischaemic attack	134
Amaurosis fugax/transient visual loss	11
**Subtotal: Cerebrovascular related events**	**210**
**Revascularisation related events**	
Heart surgery	3
Coronary angiogram	326
Non-coronary angiogram	16
Angiogram of leg/femoral angiogram	50
Renal artery angiogram	7
Carotid angiogram	5
**Subtotal: Revascularisation related events**	**407**
**Total**	**3538**

Supplementary Table S2: Comparison of pre-adjudication and post-adjudication dates

	Difference between event date pre and post-adjudication					
**Outcome**	**Exact match**	**1-7 days**	**8-30 days**	**31-90 days**	**91-180 days**	**>180 days**
Maintenance dialysis	1193 (62%)	350 (18%)	257 (13%)	89 (5%)	21 (1%)	20 (1%)
Kidney transplant	360 (72%)	130 (26%)	13 (3%)	0 (0%)	0 (0%)	0 (0%)
**Maintenance kidney replacement therapy**	**1298 (62%)**	**390 (19%)**	**260 (12%)**	**90 (4%)**	**25 (1%)**	**24 (1%)**
Non-fatal myocardial infarction	156 (73%)	30 (14%)	14 (7%)	3 (1%)	3 (1%)	8 (4%)
CHD death	87 (100%)	0 (0%)	0 (0%)	0 (0%)	0 (0%)	0 (0%)
**Any major coronary event**	**227 (76%)**	**30 (10%)**	**18 (6%)**	**4 (1%)**	**4 (1%)**	**15 (5%)**
Ischaemic stroke	156 (74%)	25 (12%)	18 (9%)	8 (4%)	1 (<1%)	2 (1%)
Unknown stroke	3 (50%)	2 (33%)	1 (17%)	0 (0%)	0 (0%)	0 (0%)
**Any non-haemorrhagic stroke**	**191 (80%)**	**20 (8%)**	**11 (5%)**	**10 (4%)**	**1 (<1%)**	**5 (2%)**
Coronary	164 (58%)	77 (27%)	21 (7%)	10 (4%)	3 (1%)	8 (3%)
Non-coronary	155 (54%)	75 (26%)	29 (10%)	13 (5%)	2 (1%)	11 (4%)
**Any revascularisation**	**300 (55%)**	**150 (28%)**	**45 (8%)**	**19 (3%)**	**6 (1%)**	**24 (4%)**
**Any major atherosclerotic event**	**597 (63%)**	**174 (18%)**	**69 (7%)**	**41 (4%)**	**12 (1%)**	**58 (6%)**

Data are n (%), with n representing the number of events within each category and percentages representing the proportion of the total number of events in each row. CHD = coronary heart disease.

Supplementary Table S3: Comparison of pre-adjudication and post-adjudication follow-up for deaths in SHARP

	Outcome after adjudication														
**Outcome before adjudication**	**CHD**	**Other cardiac**	**Isch stroke**	**Haem stroke**	**Unsp stroke**	**Other vascular**	**Cancer**	**Renal**	**GI**	**Resp**	**Other medical**	**Trauma**	**Sudden death**	**Reason unclear**	**Total**
CHD	**87 (44%)**	50 (25%)	3 (2%)	0 (0%)	0 (0%)	4 (2%)	4 (2%)	6 (3%)	6 (3%)	4 (2%)	8 (4%)	0 (0%)	17 (9%)	9 (5%)	**198**
Other cardiac	54 (11%)	**213 (42%)**	1 (<1%)	2 (<1%)	2 (<1%)	12 (2%)	11 (2%)	59 (12%)	22 (4%)	7 (1%)	23 (5%)	3 (1%)	49 (10%)	47 (9%)	**505**
Isch stroke	0 (0%)	1 (2%)	**38 (73%)**	0 (0%)	4 (8%)	1 (2%)	0 (0%)	0 (0%)	1 (2%)	0 (0%)	4 (8%)	1 (2%)	0 (0%)	2 (4%)	**52**
Haem stroke	1 (2%)	1 (2%)	2 (4%)	**36 (63%)**	10 (18%)	1 (2%)	1 (2%)	1 (2%)	0 (0%)	0 (0%)	1 (2%)	0 (0%)	1 (2%)	2 (4%)	**57**
Unsp stroke	0 (0%)	0 (0%)	2 (17%)	0 (0%)	**5 (42%)**	0 (0%)	0 (0%)	0 (0%)	0 (0%)	1 (8%)	3 (25%)	0 (0%)	1 (8%)	0 (0%)	**12**
Other vascular	2 (4%)	2 (4%)	2 (4%)	2 (4%)	2 (4%)	**29 (57%)**	2 (4%)	1 (2%)	0 (0%)	1 (2%)	2 (4%)	3 (6%)	2 (4%)	1 (2%)	**51**
Cancer	0 (0%)	0 (0%)	0 (0%)	0 (0%)	0 (0%)	1 (1%)	**190 (95%)**	2 (1%)	1 (1%)	2 (1%)	1 (1%)	0 (0%)	2 (1%)	1 (1%)	**200**
Renal	7 (3%)	9 (4%)	1 (<1%)	0 (0%)	0 (0%)	7 (3%)	6 (3%)	**136 (66%)**	6 (3%)	10 (5%)	10 (5%)	2 (1%)	7 (3%)	5 (2%)	**206**
Respiratory	5 (2%)	19 (8%)	9 (4%)	1 (<1%)	0 (0%)	5 (2%)	12 (5%)	15 (6%)	**150 (61%)**	5 (2%)	14 (6%)	4 (2%)	3 (1%)	5 (2%)	**247**
Gastrointestinal	0 (0%)	1 (1%)	1 (1%)	0 (0%)	0 (0%)	2 (2%)	9 (8%)	8 (7%)	3 (3%)	**81 (70%)**	9 (8%)	1 (1%)	0 (0%)	1 (1%)	**116**
Other medical	11 (3%)	23 (6%)	9 (2%)	5 (1%)	2 (<1%)	12 (3%)	27 (7%)	83 (21%)	23 (6%)	26 (6%)	**157 (39%)**	5 (1%)	7 (2%)	12 (3%)	**402**
Trauma or fracture	1 (2%)	2 (4%)	0 (0%)	4 (9%)	0 (0%)	1 (2%)	1 (2%)	2 (4%)	0 (0%)	0 (0%)	0 (0%)	**36 (77%)**	0 (0%)	0 (0%)	**47**
Sudden death	0 (0%)	3 (38%)	0 (0%)	0 (0%)	0 (0%)	0 (0%)	0 (0%)	0 (0%)	0 (0%)	0 (0%)	0 (0%)	0 (0%)	**5 (63%)**	0 (0%)	**8**
Reason unclear	11 (8%)	18 (13%)	3 (2%)	0 (0%)	0 (0%)	2 (1%)	12 (9%)	18 (13%)	11 (8%)	5 (4%)	10 (7%)	1 (1%)	10 (7%)	**37 (27%)**	**138**
Non-fatal event*	2 (11%)	2 (11%)	0 (0%)	0 (0%)	0 (0%)	1 (6%)	3 (17%)	6 (33%)	1 (6%)	0 (0%)	1 (6%)	0 (0%)	1 (6%)	1 (6%)	**18**
**Total**	**181**	**344**	**71**	**50**	**25**	**78**	**278**	**337**	**224**	**142**	**243**	**56**	**105**	**123**	

Percentages in parentheses are % of total number of SHARP participants with the outcome reported before adjudication. *Only includes non-fatal site-reported events that were categorised as fatal after adjudication. CHD = coronary heart disease. GI = gastrointestinal. Haem = haemorrhagic. Isch = ischaemic. Resp = respiratory. Unsp = unspecified.

Supplementary Table S4: Fact of hospitalisation, duration of hospital stay, and effect of allocation to simvastatin/ezetimibe vs placebo on refuted, unrefuted and "identified by adjudication" major atherosclerotic events

	Refuted by adjudication			Unrefuted by adjudication			Identified by adjudication of other reported events		
**Outcome**	**Hospital-ised***	**Duration of hospital stay****	**Risk ratio (95% CI)**	**Hospital-ised***	**Duration of hospital stay****	**Risk ratio (95% CI)**	**Hospital-ised***	**Duration of hospital stay****	**Risk ratio (95% CI)**
Any major coronary event	74.2%	7 (3-14)	0.97 (0.74-1.27)	98.6%	9 (5-16)	0.94 (0.75-1.18)	100%	7 (4-14)	0.88 (0.63-1.21)
Any non-haemorrhagic stroke	55.7%	9 (3-19)	0.88 (0.60-1.30)	91.5%	9 (5-17)	0.72 (0.56-0.93)	77.8%	8 (3-16)	0.85 (0.53-1.38)
Any revascularisation	82.8%	4 (2-14)	0.78 (0.53-1.15)	96.8%	7 (2-14)	0.77 (0.65-0.91)	91.8%	6 (2-14)	0.94 (0.63-1.42)
**Any major atherosclerotic event**	**70.6%**	**7 (2-14)**	**0.80 (0.65-1.00)**	**95.9%**	**7 (4-15)**	**0.80 (0.70-0.91)**	**90.8%**	**7 (3-14)**	**1.02 (0.77-1.35)**

Duration of hospital stay presented as median with interquartile range in parenthesis. Major atherosclerotic events without hospitalisation (i.e. managed as an outpatient) were assigned 0.5 days of admission. *Includes non-fatal events only. **Includes non-fatal hospitalised events only. CI = confidence interval.

Supplementary Table S5: Tabulation of major atherosclerotic events before and after adjudication in SHARP, by dialysis status

Outcome by dialysis status	Total events before adjudication	Unrefuted by adjudication	Refuted by adjudication	Identified by adjudication of other reported events	Total events after adjudication
Non-fatal myocardial infarction					
No dialysis	**207**	137 (66%)	70 (34%)	50	**187**
Dialysis	**139**	77 (55%)	62 (45%)	29	**106**
CHD death					
No dialysis	**106**	48 (45%)	58 (55%)	54	**102**
Dialysis	**92**	39 (42%)	53 (58%)	40	**79**
Any non-haemorrhagic stroke					
No dialysis	**217**	156 (72%)	61 (28%)	41	**197**
Dialysis	**123**	82 (67%)	41 (33%)	26	**108**
Coronary revascularisation					
No dialysis	**186**	168 (90%)	18 (10%)	29	**197**
Dialysis	**154**	115 (75%)	39 (25%)	40	**155**
Non-coronary revascularisation					
No dialysis	**169**	138 (82%)	31 (18%)	22	**160**
Dialysis	**187**	147 (79%)	40 (21%)	16	**163**
**Any major atherosclerotic event**					
**No dialysis**	**720**	**553 (77%)**	**167 (23%)**	**116**	**669**
**Dialysis**	**555**	**398 (72%)**	**157 (28%)**	**78**	**476**

Percentages in parentheses are % of total number of SHARP participants with the outcome reported before adjudication by dialysis status at baseline. CHD = coronary heart disease.

STROBE Statement—checklist of items that should be included in reports of observational studies

	Item No.	Recommendation	Page No.	Relevant text from manuscript
**Title and abstract**	1	(*a*) Indicate the study's design with a commonly used term in the title or the abstract	1	Title
		(*b*) Provide in the abstract an informative and balanced summary of what was done and what was found	2	Abstract
**Introduction**				
Background/rationale	2	Explain the scientific background and rationale for the investigation being reported	4	Intro para. 1
Objectives	3	State specific objectives, including any prespecified hypotheses	5	Intro final sentence
**Methods**				
Study design	4	Present key elements of study design early in the paper	5	Methods para. 1
Setting	5	Describe the setting, locations, and relevant dates, including periods of recruitment, exposure, follow-up, and data collection	5	Methods para.1 referencing of main study (10,11)
Participants	6	(*a*) *Cohort study*—Give the eligibility criteria, and the sources and methods of selection of participants. Describe methods of follow-up<br>*Case-control study*—Give the eligibility criteria, and the sources and methods of case ascertainment and control selection. Give the rationale for the choice of cases and controls<br>*Cross-sectional study*—Give the eligibility criteria, and the sources and methods of selection of participants		Methods para.1 referencing of main study
		(*b*) *Cohort study*—For matched studies, give matching criteria and number of exposed and unexposed<br>*Case-control study*—For matched studies, give matching criteria and the number of controls per case		N/A
Variables	7	Clearly define all outcomes, exposures, predictors, potential confounders, and effect modifiers. Give diagnostic criteria, if applicable	5	Methods para.1 referencing of main study and methods para.2
Data sources/ measurement	8*	For each variable of interest, give sources of data and details of methods of assessment (measurement). Describe comparability of assessment methods if there is more than one group	5	Methods para.1 referencing and methods para.2

Bias	9	Describe any efforts to address potential sources of bias	6	Methods para.2 describes blinding
Study size	10	Explain how the study size was arrived at	5	Methods para.1 referencing of main study
Quantitative variables	11	Explain how quantitative variables were handled in the analyses. If applicable, describe which groupings were chosen and why		N/A
Statistical methods	12	(*a*) Describe all statistical methods, including those used to control for confounding	6	Stats methods section (not randomized approaches used to control fully for confounding)
		(*b*) Describe any methods used to examine subgroups and interactions	6	References 10,11 for main study
		(*c*) Explain how missing data were addressed		NA
		(*d*) *Cohort study*—If applicable, explain how loss to follow-up was addressed<br>*Case-control study*—If applicable, explain how matching of cases and controls was addressed<br>*Cross-sectional study*—If applicable, describe analytical methods taking account of sampling strategy		NA
		(*e*) Describe any sensitivity analyses		NA
**Results**				
Participants	13*	(a) Report numbers of individuals at each stage of study—eg numbers potentially eligible, examined for eligibility, confirmed eligible, included in the study, completing follow-up, and analysed	7	Results para. 1
		(b) Give reasons for non-participation at each stage		N/A
		(c) Consider use of a flow diagram		N/A (flow diagram for original trial in references 10,11)
Descriptive data	14*	(a) Give characteristics of study participants (eg demographic, clinical, social) and information on exposures and potential confounders		N/A (in references 10,11)
		(b) Indicate number of participants with missing data for each variable of interest		NA
		(c) *Cohort study*—Summarise follow-up time (eg, average and total amount)	5	Para.1

Outcome data	15*	*Cohort study*—Report numbers of outcome events or summary measures over time	7	Results para.1 & 3
		Case-control study—Report numbers in each exposure category, or summary measures of exposure		N/A
		Cross-sectional study—Report numbers of outcome events or summary measures		N/A
Main results	16	(*a*) Give unadjusted estimates and, if applicable, confounder-adjusted estimates and their precision (eg, 95% confidence interval). Make clear which confounders were adjusted for and why they were included	7-8	Results para.2 & 6 and both figures
		(*b*) Report category boundaries when continuous variables were categorized		N/A
		(*c*) If relevant, consider translating estimates of relative risk into absolute risk for a meaningful time period		N/A
Other analyses	17	Report other analyses done—eg analyses of subgroups and interactions, and sensitivity analyses		Figure 2
**Discussion**				
Key results	18	Summarise key results with reference to study objectives	9	Discussion para.1
Limitations	19	Discuss limitations of the study, taking into account sources of potential bias or imprecision. Discuss both direction and magnitude of any potential bias		N/A
Interpretation	20	Give a cautious overall interpretation of results considering objectives, limitations, multiplicity of analyses, results from similar studies, and other relevant evidence	10	Discussion final paragraph
Generalisability	21	Discuss the generalisability (external validity) of the study results	9	Discussion para.2
**Other information**				
Funding	22	Give the source of funding and the role of the funders for the present study and, if applicable, for the original study on which the present article is based	11	Acknowledgements

*Give information separately for cases and controls in case-control studies and, if applicable, for exposed and unexposed groups in cohort and cross-sectional studies.

Note: An Explanation and Elaboration article discusses each checklist item and gives methodological background and published examples of transparent reporting. The STROBE checklist is best used in conjunction with this article (freely available on the Web sites of PLoS Medicine at http://www.plosmedicine.org/, Annals of Internal Medicine at http://www.annals.org/, and Epidemiology at http://www.epidem.com/). Information on the STROBE Initiative is available at www.strobe-statement.org.
